# Neuronal Polarity Pathways as Central Integrators of Cell-Extrinsic Information During Interactions of Neural Progenitors With Germinal Niches

**DOI:** 10.3389/fnmol.2022.829666

**Published:** 2022-05-04

**Authors:** David J. Solecki

**Affiliations:** Department of Developmental Neurobiology, St. Jude Children’s Research Hospital, Memphis, TN, United States

**Keywords:** germinal zone, niche, morphogen, cell polarity, Pard complex

## Abstract

Germinal niche interactions and their effect on developing neurons have become the subject of intense investigation. Dissecting the complex interplay of cell-extrinsic and cell-intrinsic factors at the heart of these interactions reveals the critical basic mechanisms of neural development and how it goes awry in pediatric neurologic disorders. A full accounting of how developing neurons navigate their niches to mature and integrate into a developing neural circuit requires a combination of genetic characterization of and physical access to neurons and their supporting cell types plus transformative imaging to determine the cell biological and gene-regulatory responses to niche cues. The mouse cerebellar cortex is a prototypical experimental system meeting all of these criteria. The lessons learned therein have been scaled to other model systems and brain regions to stimulate discoveries of how developing neurons make many developmental decisions. This review focuses on how mouse cerebellar granule neuron progenitors interact with signals in their germinal niche and how that affects the neuronal differentiation and cell polarization programs that underpin lamination of the developing cerebellum. We show how modeling of these mechanisms in other systems has added to the growing evidence of how defective neuronal polarity contributes to developmental disease.

## General Germinal Zone Considerations

Neuronal progenitor cells and neural stem cells residing in germinal zones (GZs) throughout the central nervous system face a bewildering array of extracellular signals that are critical to controlling decisions such as how many more progeny to produce or when to exit the cell cycle and terminally differentiate (Choi et al., 2005; Corbin et al., 2008; Bjornsson et al., 2015; Dehay et al., 2015; Ortega et al., 2018). Among these signals, diverse secreted morphogens, such as hedgehogs and Wnts or extracellular matrix (ECM) molecules, are made in an autocrine or paracrine manner by progenitors and neural stem cells themselves or by supporting cells such as glia or endothelia in niche environments (Borello and Pierani, 2010; Kazanis and ffrench-Constant, 2011; Tiberi et al., 2012; Barros et al., 2020). Moreover, homotypic and heterotypic cell-to-cell contacts have dual roles, being involved in quorum sensing between cell types and serving as critical anchor points for migration or cell sorting events (Marthiens et al., 2010; Solecki, 2012; Famulski and Solecki, 2013; Morante-Redolat and Porlan, 2019).

Each GZ and niche environment has a unique complement of these genetically encoded secreted proteins or cell-recognition mechanisms that corresponds precisely to the required output of that particular niche. For example, the rapid development of the mouse cerebral cortex corresponds to a GZ extrinsic code that promotes the rapid elaboration of the neurons that populate each layer of the cortical plate with pyramidal neurons (Qian et al., 1998, 2000; Corbin et al., 2008; Uzquiano et al., 2018), whereas the adult subventricular zone (SVZ) vascular niche favors the maintenance of quiescent stem cells that sporadically produce new neurons throughout the life of the rodent (Shen et al., 2008; Tavazoie et al., 2008). Each decision made by the cells in these specialized niches involves an intricate balance between the reception of extrinsic morphogen signals and the cell-intrinsic mechanisms by which the signals are interpreted to transform morphogenic information into executable cell biological programs that ultimately underlie circuit formation. Despite our progress in identifying genetically encoded morphogens and the fundamental decisions they control, how cell-intrinsic machinery translates morphogen information into consolidated cell biological programs remains one of the most elusive aspects of neural development because of the difficulties in determining how cells integrate diverse pathways in time and space.

## The Early Postnatal Cerebellar Germinal Zone and Lamination

Cerebellar granule neurons (CGNs) are prototypical model systems that have enabled researchers to make inroads into understanding how signals are integrated with cell biological programs, especially in the context of signaling cascades that control GZ exit and the onset of neuronal differentiation (Hatten and Roussel, 2011; Singh and Solecki, 2015; Leto et al., 2016; Iulianella et al., 2019; Consalez et al., 2020). The GZ of the developing mouse cerebellum, particularly the external germinal layer (EGL), which gives rise to granule neurons, is unique among brain regions in that (1) the nearly crystalline structure of the developing cerebellar layers and their cellular composition has been exhaustively examined at both the light and electron microscopy levels and (2) cerebellar investigators have unprecedented access to almost unlimited numbers of granule neurons for *in vivo*, *ex vivo*, and *in vitro* experimentation at both the tissue and single-cell levels. This combination of the fundamental ground truth of how the GZ is structured and deep access to cerebellar granule neuron manipulation has led to the mouse cerebellar GZ being one of the best characterized GZs in the central nervous system.

After arising from radial glial neural stem cells of the rhombic lip, CGN progenitors (GNPs) migrate to the EGL secondary GZ on the surface of the cerebellar anlage (Ryder and Cepko, 1994; Wingate and Hatten, 1999). Massive GNP proliferation in the EGL *via* mostly symmetric cell divisions, with cell cycles faster than those of cell lines dividing *in vitro*, creates a cohort of CGNs that not only account for approximately 85% of all cerebellar neurons but also represent the most abundant neuronal type in the entire brain (Fujita, 1967; Espinosa and Luo, 2008; Roussel and Hatten, 2011). After GNP terminal differentiation, newly formed CGNs are displaced slightly inward from the outermost layer of the EGL (oEGL) to the inner EGL (iEGL), where they extend parallel fibers, migrate tangentially along the axons of CGNs that have already differentiated, and fasciculate with their differentiated neighbors. Approximately 36 h after the final division of their GNP parent cell, CGNs are ready to move to their final destination in the internal granule layer (IGL) by radially migrating as single cells along Bergmann glial fibers past the Purkinje cell layer.

## Niche Factors Promoting GNP Proliferation and Differentiation

This section summarizes the molecular participants in cell-to-cell communication events that control the output of GNP proliferative decisions and the elaboration of the CGN differentiation programs during the critical steps in CGN development. Pioneering studies by Gao and Hatten using a GNP and CGN culture system showed that growth factors such as IGF1, bFGF, and EGF, which are potent mitogens, sensed by receptor tyrosine kinases, for neural stem cells throughout the brain, elicit only minimal enhancement of GNP proliferation (Gao et al., 1991). In contrast, GNPs grown in culture at high density in cellular reaggregates stimulated proliferation that was nearly 10-fold higher than that seen in control cultures, implying that GNP homotypic interactions among progenitor cells are essential to maintain progenitor cell divisions within the densely packed EGL. Paracrine interactions are also critical modulators of GNP neurogenesis in the oEGL niche. Now-classic experiments showed that Purkinje cells secrete the Sonic hedgehog (Shh) morphogen, which diffuses over long distances to GNPs residing in the oEGL and is the most potent mitogen discovered for these cells to date (Dahmane and Ruiz i Altaba, 1999; Wallace, 1999; Wechsler-Reya and Scott, 1999; Lewis et al., 2004). Shh control of GNP proliferation is evolutionarily conserved, as genetic lesions that activate the Shh pathway in both humans and mice lead to transformation of GNPs and ultimately to the formation of medulloblastoma tumors. The oEGL niche contains undefined constituents that support Shh-induced GNP neurogenesis, as GNPs seeded onto thick cerebellar slices in an overlay assay respond to Shh only when they settle in the oEGL (Choi et al., 2005). In addition to Purkinje cells, the meningeal fibroblasts that overlie the oEGL also provide short-range paracrine signals that modulate GNP proliferation. Early electron microscopy studies demonstrated that GNPs maintain contact with the basal lamina produced by meningeal fibroblasts (Hausmann and Sievers, 1985), and chemical ablation of the meninges leads to reduced GNP proliferation (von Knebel Doeberitz et al., 1986). The meninges express several molecules that are arrayed near the oEGL niche and can directly affect GNP proliferative outcomes. One of these molecules is Jagged1, which activates GNP Notch2 receptors, ultimately initiating a transcriptional cascade that maintains GNPs in the undifferentiated state (Solecki et al., 2001). Meningeal fibroblasts also produce stromal-derived factor 1 (SDF-1) (Zou et al., 1998; Zhu et al., 2002), which synergizes with Shh at the level of GNP proliferation (Klein et al., 2001). Finally, as discussed in detail below, the meningeal secreted extracellular matrix, the main constituent of the basal lamina contacted by GNPs, also modulates GNP responsiveness to Shh.

Like GNP proliferation, CGN differentiation is controlled by a combination of autocrine or paracrine interactions in the niche that drive progenitors to the postmitotic state. Co-culture assays with highly purified populations of CGNs and cerebellar glia showed that interactions between GNPs and CGNs or between GNPs and Bergmann glia drive progenitors into the postmitotic state (Gao et al., 1991). Follow-up studies showed that CGNs drive GNP differentiation via surface expression of vitronectin (Pons et al., 2001), an ECM component in the iEGL, and secretion of bone morphogenic proteins 2 and 4 (Bmp2/4) (Rios et al., 2004). Both factors inhibit GNP Shh signaling. Cerebellar glia produces basic fibroblast growth factor (bFGF), which stimulates CGN axon extension (Hatten et al., 1988), and N-cadherin, which facilitates CGN migration, two processes concurrent with CGN differentiation (Horn et al., 2018). Finally, Purkinje cells produce at least three secreted signals that promote CGN maturation: brain-derived neurotrophic factor (BDNF) (Schwartz et al., 1997), Wnt3 (Anne et al., 2013), and pituitary adenylate cyclase–activating peptide (PACAP) (Nicot et al., 2002; Niewiadomski et al., 2013). Wnt3 and PACAP both act by inhibiting Shh-dependent GNP proliferation to drive CGNs into the postmitotic state.

## Cell Polarity and the Intrinsic Machinery That Interprets Germinal Niche Signals

Despite the discovery of the extensive array of molecules that activate the signaling cascades modulating GNP proliferation and CGN differentiation, few cell-intrinsic mechanisms for integrating the reception of such signals with the cell biological mechanisms elaborated during differentiation have been characterized. Cell polarity represents a promising cell-intrinsic mechanism by which to coordinate tissue information with the internal organization of the cell during a morphogenic program (Singh and Solecki, 2015; Laumonnerie and Solecki, 2018). In the classic example of epithelial cells, polarity signaling cascades such as the partitioning-defective (Pard) signaling complex and planar cell polarity signaling cascades consistently orient cells in the tissue so that the polarity axes align (Goldstein and Macara, 2007; Baum and Georgiou, 2011; Campanale et al., 2017). In the case of apical–basal polarity, polarity signaling enforcement of a consistent epithelial orientation synchronizes the transport function of epithelial cells across epithelial tissues and ensures tissue function. Polarity signaling similarly coordinates the structure and function of neural tissues. For example, the apical–basal polarity of radial glial cells ensures the appropriate lamination of cortical regions of the brain (Chou et al., 2018), whereas the axodendritic polarity of neurons controls proper information flow in neuronal circuits (Barnes et al., 2008). Polarity signaling from the Pard complex also plays critical roles in the early stages of neuronal differentiation, such as the timing of GNPs becoming postmitotic and the onset of CGN GZ exit in the developing cerebellum (Laumonnerie and Solecki, 2018). Three main components of the Pard complex, Pard3, Pard6α, and Prkcz, are expressed at low levels in GNPs, increase their expression dramatically in differentiating CGNs, and are necessary for CGN differentiation (Famulski et al., 2010; Singh et al., 2016). Pard3 and Pard6α gain of function in GNPs, which generally express low levels of these proteins, stimulates terminal differentiation and GZ exit by encouraging differentiation-specific cytoskeletal organization and junctional adhesion molecule C (JAM-C) adhesion to differentiated CGNs and Bergmann glia. Low levels of Pard3 and Pard6α expression in GNPs represent an active developmental cell polarity switch, because GNPs express an E3 ubiquitin ligase, seven *in absentia* homolog 2 (Siah2) (Famulski et al., 2010), and a transcriptional repressor, zinc finger E-box–binding homeobox 1 (Zeb1), that act as complementary Pard complex inhibitors that enforce GNP GZ occupancy (Singh et al., 2016). As GNPs differentiate, Siah2 and Zeb1 expression recedes, leading to enhanced Pard complex–driven cytoskeletal organization and JAM-C adhesion that drives GZ exit and radial migration initiation.

**FIGURE 1:**
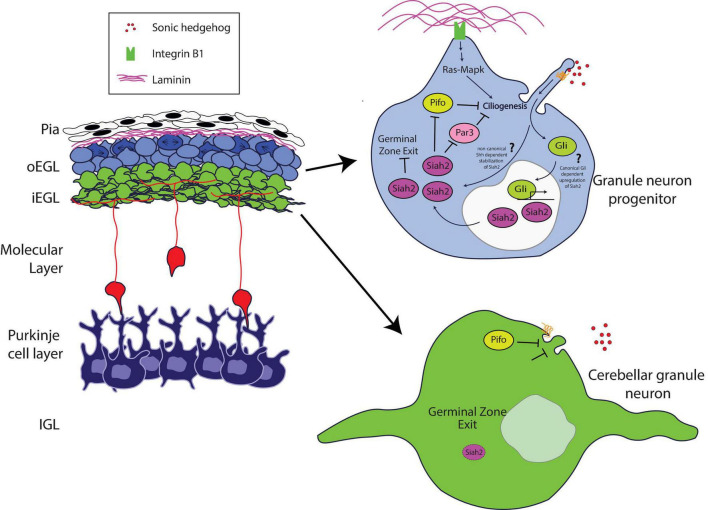
Schematic of how Siah2 is regulated in developing CGNs. The laminin-rich basement membrane surrounding the oEGL in a developing cerebellum promotes Shh signaling driven primary ciliogenesis in GNPs via Integrin β1—Ras/Mapk signaling. The primary cilium allows GNPs to sense the Shh mitogen and activate the Shh pathway to maintain Siah2 expression which in turn promotes GZ occupancy by inhibiting GZ exit. Siah2 acts in a feed-forward mechanism to maintain mitogen sensitivity by promoting primary ciliogenesis through the antagonism of a key cilia disassembly proteins Pifo and Dbn, and the polarity inducer Pard3. As GNPs leave the oEGL, the lack of trophic support leads to the disassembly of the primary cilium and loss of sensitivity to Shh, which promotes CGN differentiation.

Recent studies have expanded our knowledge of the role of the Pard complex in mediating key integrative steps in the response of GNPs and CGNs to niche conditions to organize cell biological pathways responsible for differentiation. Ong et al. (2020) used sophisticated imaging technologies to reveal how the Pard complex participates in a coincidence detection circuit between the pial ECM and Shh signaling at the level of GNP ciliogenesis (Figure 1). Pioneering studies by Mueller and colleagues showed that Shh signaling required beta 1 integrin receptors to modulate GNP proliferation effectively; however, the mechanism by which this occurred was unclear (Blaess et al., 2004). Ong et al. showed that Ras signaling stimulated by integrin receptor binding to pial secreted laminin activates the expression of Siah2 in a manner that requires Shh signaling, which ultimately maintains GNPs in the proliferative state.

How do niche signals cooperate to regulate GNP proliferation? Cutting-edge three-dimensional electron microscopy that enabled full volumetric reconstruction of single cells within the intact oEGL and iEGL niche environments showed that GNPs are more ciliated than are CGNs. Cilia containing the Patched and Smoothened receptors are the primary sites within Shh-responsive cells that transduce the signaling cascade for this morphogen (Huangfu and Anderson, 2005; Caspary et al., 2007; Rohatgi et al., 2007). Complex epistasis experiments involving Siah2, Ras, and integrin receptors revealed the molecular basis for this difference in ciliation. Siah2 regulates GNP Shh responsiveness in a feed-forward fashion by maintaining GNP primary cilia in an integrin-dependent and Ras-dependent manner (Ong et al., 2020). Analysis of Siah2 ubiquitination targets defined a novel role for the Pard complex in promoting GNP differentiation. By using Siah2 gain of function as the basis for a live cell–imaging target rescue screen that is possible only with the large number of GNPs present in the developing cerebellum, Ong and colleagues revealed that Pard3 expression causes cilia retraction. Therefore, when coincidence detection between ECM and Shh signals in the oEGL niche predominates, the resulting Ras-dependent Siah2 activity diminishes the ability of Pard3 to facilitate cilia retraction, leading to the maintenance of Shh responsiveness. However, when Pard3 expression is elevated in the iEGL, a cell biological program promoting cilia retraction is favored, leading GNPs to be less sensitive to the Shh mitogen and allowing their transition to the differentiated CGN state.

**FIGURE 2:**
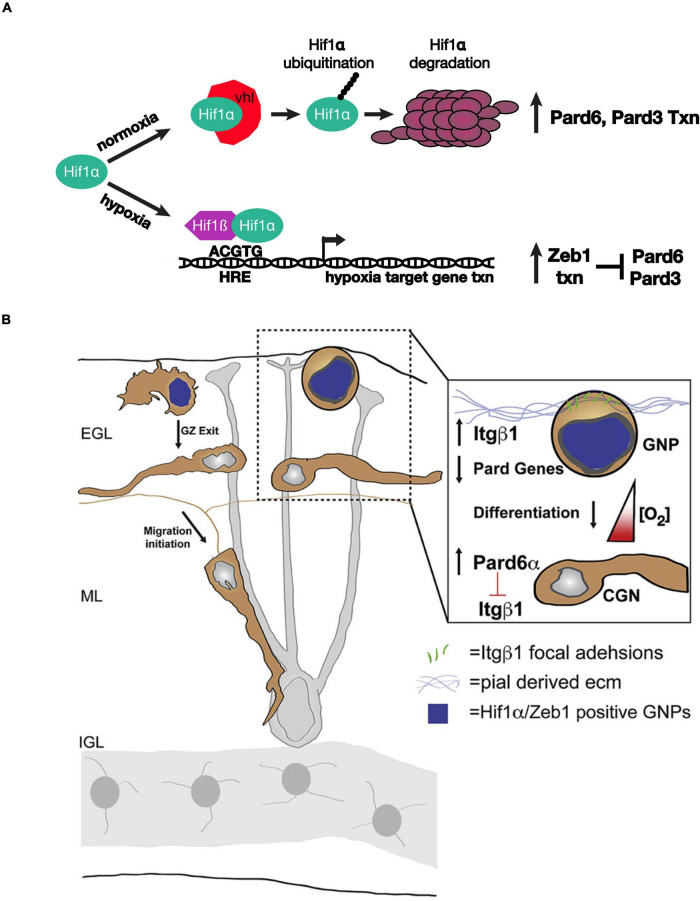
Schematic of how oxygen tension regulates neuronal polarity in developing CGNs. **(A)** Model for the genetic interactions between Hif1α, Zeb1, and the Pard proteins in hypoxia or normoxia. **(B)** The laminin-rich basement membrane surrounding the oEGL in a developing cerebellum. GNPs expressing Hif1α and Zeb1 (blue nuclei) have lower level of Pard6α gene expression. As oxygen levels increase during development Pard6α gene expression increases which loosen Itgβ1 adhesion to the pial basal lamina. Images adapted from Kullmann et al. (2020) with permission from (Elsevier).

Kullmann et al. (2020) used an array of advanced light-sheet imaging techniques to demonstrate a unique interaction between the Pard complex and oxygen tension, a non-genetically encoded niche condition that controls the timing of GNP differentiation (Figure 2). Macro light-sheet imaging and machine learning quantitation of the vasculature of iDISCO-cleared developing cerebellum revealed an interesting correlation with CGN differentiation. The EGL and molecular layer of postnatal day 7 cerebella are poorly vascularized when compared with the IGL, where differentiated CGNs reside, and with neighboring regions of the brain, where neurons differentiate earlier than in the cerebellum, suggesting that the cerebellar niche is an oxygen-poor environment. This hypothesis was bolstered by high hypoxyprobe staining of these layers in the developing cerebellum and high levels of hypoxia-inducible factor 1 alpha (Hif1α) in GNPs during the stages of cerebellar development with low vascularization. Hif1α, which is negatively regulated in normoxia by the von Hippel–Lindau (VHL) tumor suppressor protein, a component of an E3 ubiquitin ligase complex (Gossage et al., 2015), is not only a marker for hypoxia but also an evolutionarily conserved transcription factor that activates the expression of genes that are activated in response to hypoxia (Kaelin and Ratcliffe, 2008; Ivan and Kaelin, 2017). Genetic deletion of Hif1α and VHL *in vivo* revealed that the Hif1α pathway enforces GNP occupancy with the EGL niche and delays the timing of CGN migration initiation. Hif1α binds to the *Zeb1* gene promoter and activates Zeb1 mRNA expression. As Zeb1 transcriptionally represses Pard3 and Pard6α mRNA expression in GNPs, epistasis studies were needed to determine whether hypoxia or the Hif1α pathway enforced GZ occupancy by inhibiting the Pard complex gene expression. GZ occupancy stimulated by Hif1α gain of function, or hypoxia, could be rescued equally by Zeb1 loss of function and by Pard complex gain of function, showing for the first time that oxygen tension in the EGL regulates the onset of CGN polarization directly via Pard3 and Pard6a expression.

How does Hif1α - Zeb1 antagonism of Pard complex function enforce GZ occupancy? Lattice light-sheet structured illumination microscopy (LLSM-SIM) was instrumental in determining the precise cellular mechanism (Chen et al., 2014). Electron microscopic studies showed that GNPs maintain contact with the basal lamina during their time in the GZ niche (Hausmann and Sievers, 1985); however, imaging of GNP focal adhesion to pial ECM was not possible with conventional light microscopy because of the poor signal ratio and resolution. LLSM-SIM revealed that GNPs maintained in the undifferentiated state by over-expressing Hif1α or Zeb1 have numerous ECM focal adhesions and that Pard6α expression potently diminishes these adhesions, probably at the transcriptional level. Integrin receptors are a central component of the focal adhesions that recognize ECM. Not only does deleting beta 1 integrin rescue hypoxia-induced GZ occupancy, but elevating beta 1 integrin expression maintains GNPs in their germinal niche. Taken together, these findings show that the environmental niche conditions modulate how GNPs interact with ECM landmarks within the niche via Hif1α or Zeb1 inhibition of neuronal polarization. Moreover, the two studies highlight how Siah2-dependent post-translation regulation of the Pard complex converges upon the same integrin receptors that are regulated by the Hif1α and Zeb1 pathways in response to oxygen tension.

## Relevance to Other Models and to Neurodevelopmental Disease

Polarity regulation mechanisms discovered in the mouse cerebellum may have relevance to the mouse cerebral cortex. For example, Zeb1 has been found to regulate cortical neuron differentiation *via* polarity gene expression in a manner similar to that reported in CGNs (Jiang et al., 2018; Liu et al., 2019; Wang et al., 2019). In the case of the cortex, Zeb1 must bind to CTBP2 to suppress NeuroD1 expression at the developmental stage between radial glia and intermediate progenitors, which suggests that the NeuroD1 basic helix–loop–helix (bHLH) transcription may be upstream of polarity gene expression. Interestingly, prolonged Zeb1 expression in the cerebral cortex leads to subcortical band heterotopia, suggesting that the Zeb1 polarity gene regulatory pathways are involved in neuronal migration disorders distinct from those induced by defective cytoskeletal genes.

Although few mutations in polarity genes have been observed in human neurodevelopmental disorders, there is growing evidence that polarity pathways are, nevertheless, perturbed in human disease. In humans, medulloblastoma comprises a spectrum of pediatric brain tumors derived from the transformation of progenitor cells in the major GZs of the cerebellum. GNPs have been shown definitively to be the cell type of origin for Shh-class medulloblastomas (Goodrich et al., 1997; Kim et al., 2003; Oliver et al., 2005; Rohatgi et al., 2007; Yang et al., 2008). These tumors express elevated levels of Zeb1 and Siah2 and low levels of Pard complex (Singh et al., 2016; Ong et al., 2020), consistent with the polarity trajectories described for mouse GNPs. Genetic deletion of the Patched1 Shh receptor, which stimulates Shh medulloblastoma formation in humans, creates a cohort of GNPs that do not leave the EGL GZ in mouse models of Shh medulloblastoma. In these mouse models, manipulating polarity pathways in pre-tumorigenic GNPs revealed that elevating the level of Pard complex or reducing Zeb1 or Siah2 expression restores appropriate GZ exit and CGN differentiation. Therefore, differentiative therapy of pediatric brain tumors by promoting neuronal polarization may be a promising treatment strategy.

The Zeb1–Hif1α–Pard complex pathway may also be relevant for neurodevelopmental disorders related to prenatal health problems. Intrauterine growth restriction (IUGR) affects many pregnancies, leading to hypoxia in developing brain tissue that ultimately causes motor and cognitive defects in affected children. Among the more prominent features associated with IUGR is defective cerebellar development. A recent study using a porcine model of human neurodevelopment linked Pard complex defects directly to IUGR (Iskusnykh et al., 2021). IUGR in piglets leads to an enlarged EGL and defective GZ exit of GNPs, as assayed by *ex vivo* slice preparations of postnatal pig cerebella. Prominent reductions in Pard3 and Jam-C mRNA expression in the cerebella of piglets with IUGR suggested that GZ exit defects were due to defective neuronal polarization. Indeed, an *ex vivo* GZ exit assay like those developed with the mouse cerebellum showed that restoring Pard3 and Jam-C expression rescued GZ exit in IUGR cerebella to control levels. Interestingly, that study showed that Pard3 and Jam-C are also required for appropriate survival of differentiated CGNs, suggesting functions for neuronal polarity beyond the migration step in cerebellar development. Although the study did not link elevated prenatal hypoxia to defective GZ occupancy, the findings of Kullmann et al. (2020) suggest that the hypoxia associated with IUGR positions Hif1α and Zeb1 as central mediators of the reduced polarity gene expression observed when uterine insufficiency leads to prenatal hypoxia. These findings raise the tantalizing possibility of elevated polarity signaling having therapeutic benefits for two unrelated classes of neurological disorders: pediatric cancers and defects in brain development associated with prenatal hypoxia.

## Author Contributions

The author confirms being the sole contributor of this work and has approved it for publication.

## Conflict of Interest

The author declares that the research was conducted in the absence of any commercial or financial relationships that could be construed as a potential conflict of interest.

## Publisher’s Note

All claims expressed in this article are solely those of the authors and do not necessarily represent those of their affiliated organizations, or those of the publisher, the editors and the reviewers. Any product that may be evaluated in this article, or claim that may be made by its manufacturer, is not guaranteed or endorsed by the publisher.
